# Correntropy-Induced Discriminative Nonnegative Sparse Coding for Robust Palmprint Recognition

**DOI:** 10.3390/s20154250

**Published:** 2020-07-30

**Authors:** Kunlei Jing, Xinman Zhang, Guokun Song

**Affiliations:** 1 School of Automation Science and Engineering, Faculty of Electronic and Information Engineering, MOE Key Lab for Intelligent Networks and Network Security, Xi’an Jiaotong University, Xi’an 710049, China; zhangxinman@mail.xjtu.edu.cn; 2 Sichuan Gas Turbine Research Institute of AVIC, No. 6 Xinjun Road, Xindu District, Chengdu 610500, China; 2014223020072@stu.scu.edu.cn

**Keywords:** robust palmprint recognition, regression analysis, correntropy metric, discriminative nonnegative regularizer, nonnegative constraint, constrained particle swarm optimizer

## Abstract

Palmprint recognition has been widely studied for security applications. However, there is a lack of in-depth investigations on robust palmprint recognition. Regression analysis being intuitively interpretable on robustness design inspires us to propose a correntropy-induced discriminative nonnegative sparse coding method for robust palmprint recognition. Specifically, we combine the correntropy metric and *l*_1_-norm to present a powerful error estimator that gains flexibility and robustness to various contaminations by cooperatively detecting and correcting errors. Furthermore, we equip the error estimator with a tailored discriminative nonnegative sparse regularizer to extract significant nonnegative features. We manage to explore an analytical optimization approach regarding this unified scheme and figure out a novel efficient method to address the challenging non-negative constraint. Finally, the proposed coding method is extended for robust multispectral palmprint recognition. Namely, we develop a constrained particle swarm optimizer to search for the feasible parameters to fuse the extracted robust features of different spectrums. Extensive experimental results on both contactless and contact-based multispectral palmprint databases verify the flexibility and robustness of our methods.

## 1. Introduction

Biometrics, like face, fingerprint, and iris images, have been exhaustively investigated for identity verification [1]. With lower risk of forgery, richer texture, and more comfortable acquisition mode, compared with face, fingerprint, and iris images, palmprints have drawn significant attention gradually [2]. Palmprint recognition methods can be roughly divided into categories [3] such as texture modeling-based [4,5,6,7,8,9], subspace learning-based [10,11,12,13], and local descriptor-based [14,15,16,17,18]. These three categories of methods attempt to extract critical features by ideally defined transformations, principal directions, or descriptors. However, on the one hand, their feature extraction approaches relying on fine prior knowledge of texture location do not apply to diverse scenarios. On the other hand, some feeble but valuable wrinkles are abandoned. What’s more, despite a little work that merely considers palmprint image degeneration due to the objective rotation and illumination variation [15,19], most of the methods neglect to consider robust palmprint recognition because of the potential occlusion and corruption in real-world applications.

### 1.1. Research Actuality

Recent decades have witnessed the fruitful findings of robust recognition on other biometrics, among which regression analysis has aroused the most attention for its intuitive interpretability of robustness design [20]. Compared with the mainstream palmprint recognition methods, the regression-based methods extract features without relying on the prior knowledge of texture location, and all the valuable pixels are used in its vector-wise operation. It seems we can draw some inspirations from the regression-based methods to realize robust palmprint recognition.

The linear regression classifier (LRC) may be one of the foremost methods in regression-based biometric recognition, which seeks suitable representation coefficients of a query sample and classifies it by examining which class can lead the minimal reconstruction residual [21]. With the *l*_1_-norm regularization, the sparse representation classifier (SRC) showed impressive performance on biometric recognition [22]. Zhang et al. claimed that it was the collaboration mechanism of the *l*_1_-norm that rendered SRC resultful and replaced the *l*_1_-norm with the *l*_2_-norm to put forward a collaborative representation classifier (CRC) [23]. Huang et al. introduced the *l*_2,1_-norm to achieve both flat and structured sparse coding [24]. Moreover, Xu et al. created a novel regularization to propose a discriminative SRC (DSRC) [25]. The regularization-based methods utilized the *l*_2_-norm or *l*_1_-norm to measure the representation errors under the assumption that the errors follow a Gaussian or Laplacian distribution [20]. Such a simplified treatment is capable of handling some simple corruptions, but could be unreasonable when facing more complicated contaminations such as dense corruption and gross occlusion.

To alleviate the impact caused by contaminations, Wright et al. introduced an augmented dictionary into SRC to create a robust SRC (RSRC) [22]. By extracting the centroids and variation of the training samples, Deng et al. proposed a superposed SRC (SSRC) [26]. Although these ideas improved the representation ability of the dictionaries, they can not overcome the drawback of the regularization-based methods, which leads to their limited robustness. To characterize the representation errors, Yang et al. [27,28] proposed the robust sparse coding (RSC) and regularized robust coding (RRC), respectively. Drawing ideas from the information theory, He et al. measured the errors by the correntropy-based sparse representation (CESR) [29]. These error detection-based methods yielded promising results to continuous occlusion, but they can be easily trapped by the undetected errors when the occlusion is heavy [30]. The nuclear norm-based matrix regression (NMR) method appealed to model the low-rank structure of the representation errors [30,31]. Whereas, the low-rank modeling is unrealistic in practice when samples are subjected to disperse corruption. Recently, the half-quadratic (HQ) method and Laplacian-uniform mixture-driven iterative robust coding (LUMIRC) method were proposed for error detection and correction [32,33]. However, both of them neglected the fact that the robustness of the regression-based methods relies not only on the error estimator but also on the sparsity regularizer.

All the work analyzed above has a common intention of attempting to get rid of the flawed entries in the contaminated sample and obtain promising recognition performance with the partial pure entries [19,20,21,22,23,24,25,26,27,28,29,30,31,32,33,34]. However, when the features of different classes are similar, partial information is insufficient to support us to correctly distinguish one class from the others. Fortunately, multimodal biometrics acquired from multi-views can provide more useful features to address this problem. Taking multispectral palmprint images for example. Samples acquired under different spectrums can provide more information against the pixel loss caused by contaminations [35]. Up to now, much efforts have been made for multimodal biometric recognition by exploiting summation, wavelet transform, and competitive coding to fuse the features of different modes [36,37,38]. However, insightful explanations of why these strategies make effects were missed, and the potential contaminations were not considered.

### 1.2. Motivations and Contributions

In view of the merits and demerits of all the aforementioned studies, either the regularization-based or error detection-based work can only handle a specific contamination case, i.e., corruption or occlusion. We expect to obtain a flexible robust scheme against various contaminations in real-world applications. 

Correntropy was demonstrated to be particularly robust to non-Gaussian noises and large outliers was successfully applied for feature selection and signal processing [39,40]. Compared with the methods in [28,31,32,33] that detected errors in a heuristic way, correntropy provided a realistic metric approach that was theoretically promised to have desirable measure properties and approximate solution by the information theory and HQ optimization theory [40]. 

As was demonstrated in [22], the sparsest representation prefers to express a query sample with its homologous samples. If the representation coefficients are not sparse enough, elements corresponding to the inhomogeneous samples of the query sample will emerge. Then, the coding errors will contain the difference among diverse classes and could not reflect the real contamination anymore, which would greatly degenerate the error estimator. Since exploring the discriminability among the training samples encourages the sparsity of the representation coefficients [25], we argue that discriminative sparse coding is conductive to precise error estimation (see the verifications in Section 4). 

In addition, the conventional sparse representation expresses the query sample with a combination of the dictionary samples, which involves both additive and subtractive operations. In the sparse coefficients, the emerging negative elements are not only trivial and meaningless, but also can lead the extracted features to ‘cancel each other out’. This is contrary to the intuitive notion of combining samples into a whole and the intention of extracting significant intra-class features for reliable classification [41]. Other arguments for nonnegative coding arise from biological modeling and hyperspectral image decomposition, where the sparse representation coefficients are required to be nonnegative [42,43].

**Figure 1:**
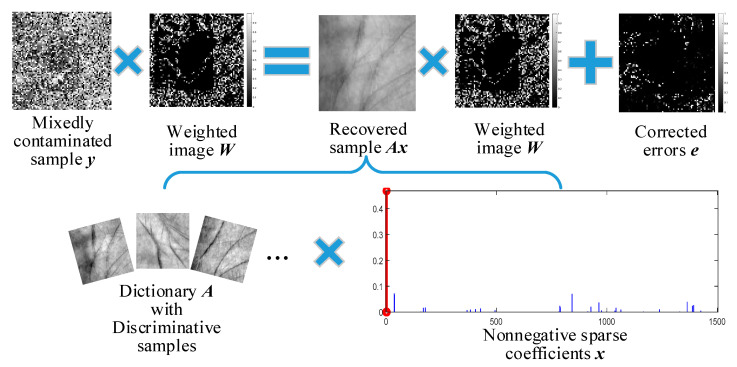
Regression-based CDNSC. The mixedly contaminated query sample can be expressed as a linear combination of the weighted discriminative dictionary samples plus the corrected errors.

Inspired by the above analyses, a cooperative error estimator (CEE) composed of a correntropy-induced error detector and a sparse error corrector is designed. Moreover, we combine CEE with a tailored discriminative nonnegative sparse regularizer (DNSR) to propose a joint scheme, named correntropy-induced discriminative nonnegative sparse coding (CDNSC), to cope with corruptions, occlusions, and the mixture of them. We also explore a feasible feature fusion strategy to extend CDNSC to robust multispectral palmprint recognition. Figure 1 illustrates the core idea of CDNSC. 

Given a query sample with mixed-contaminations, the correntropy metric detects the errors via a weighted image, while the *l*_1_-norm corrects the undetected ones. Meanwhile, DNSR produces discriminative nonnegative sparse coding to stimulate CEE to precisely estimate errors. Thus, we obtain significant features (corresponding to the red line in Figure 1) of the query sample. The extensive experimental results in Section 5 show that our algorithm outperforms all the selected state-of-the-art methods in all challenging cases, where the variation of illumination and posture, corruptions, and two types of occlusion are all considered. Our contributions are summarized as follows:

- The correntropy metric and *l*_1_-norm are combined to compose an error estimator for cooperative error detection and correction. We further equip the estimator with a discriminative nonnegative sparse regularizer to propose CDNSC to address various contaminations, like dense corruption, gross occlusion, and the mixture of them.
- To obtain the analytical solution of the unified scheme, we propose an efficient method to address the nonnegative constraint, namely, converting it into a nontrivial equality constraint. Then, with some self-developed skills, the new nondifferentiable equality constraint problem is expressed with a continuous formulation. Thus, combined with half-quadratic optimization, a reweighted alternating direction method of multipliers (ADMM) can be derived to obtain the closed-form solution of the reformulated problem.
- The proposed CDNSC is extended for robust multispectral palmprint recognition. We develop a constrained particle swarm optimizer to search for the feasible parameters to fuse the extracted robust features of different spectrums. This provides a new idea for extending the single-mode biometric recognition methods to multimodal biometric recognition.

The remainder of this paper is organized as follows: Section 2 reviews the researches on coding regularization, non-negative sparse representation, and error estimation. Section 3 introduces CEE, DNSR, the optimization of CDNSC, and its expansion for multispectral palmprint recognition. Section 4 analyzes the effectiveness of CDNSC. Section 5 carries out experimental verifications. Section 6 concludes this paper.

## 2. Related Work

In the following content, we will use bold symbols to signify matrix or vector variables and normal symbols to signify their elements. Given a dictionary $A\in {\mathbb{R}}^{D\times L}$ containing *L* vectorized *D*-dimensional training samples of diverse classes, the regression-based methods explore appropriate coefficients $x\in {\mathbb{R}}^{L}$ to facilitate the subsequent classification by representing a vectorized query sample $y\in {\mathbb{R}}^{D}$ with a linear reconstruction Ax.

### 2.1. Coding Regularization

SRC gets sparse coding ***x*** over dictionary A by employing the *l*_0_-norm. The *l*_0_ minimization is NP-hard and equals to the *l*_1_-regularized minimization as long as ***x*** is sparse enough [44]. To deal with the contaminations, the constraint y=Ax is relaxed to:

(1) $$\min{\left\|x\right\|}_{1}\;s.t.\;{\left\|y-Ax\right\|}_{2}^{2}\leq e$$

where ${\left\|x\right\|}_{1}=\sum_{i=1}^{L}\left|x_{i}\right|$ is the *l*_1_-norm, $x_{i}$ denotes the i-th element of variable x, and e denotes the coding errors. Problem (1) is the classical Lasso [45] that can be solved by leveraging the least angle regression (LAR) [46]. To better deal with the contaminations, Wright et al. further introduced an augmented dictionary into SRC to propose RSRC [22]:

(2) $$\min{\left\|x\right\|}_{1}\;s.t.\;y=\bar{A}x$$

where $\bar{A}=\left[A,I\right]$, and I is an identity matrix to fit the corruption. With a novel regularizer, DSRC presented an efficient discriminative sparse coding method [25]:

(3) $$\underset{x}{\min}{\left\|y-Ax\right\|}_{2}^{2}+\gamma \sum_{i=1}^{L}\sum_{j=1}^{L}{\left\|A_{:i}x_{i}+A_{:j}x_{j}\right\|}_{2}^{2}$$

where $A_{:i}$ is the i-th column of dictionary A, and γ is a tunable parameter.

Regularizers in Equations (1)–(3) benefit to defend the robustness by selectively extracting sparse features. However, when the complicated corruptions occur, the *l*_2_-norm is improper to measure the coding errors anymore.

### 2.2. Nonnegative Sparse Representation

An essential issue of SRC is to explore an interpretable nonnegative sparse coding x, with which a query sample is reconstructed by only addition [43]. The nonnegative matrix factorization (NMF) is an important technique to find such coefficients. Given dictionary A, NMF aims to find two nonnegative matrixes $U=\left[u_{ik}\right]\in {\mathbb{R}}^{D\times R}$ and $V=\left[v_{ik}\right]\in {\mathbb{R}}^{L\times R}$ that:

(4) $$\underset{U,V}{\min}{\left\|A-UV^{T}\right\|}_{2}^{2}=\underset{u_{ik},v_{jk}}{\min}\sum_{i=1}^{D}\sum_{j=1}^{L}{\left(a_{ij}-\sum_{k=1}^{R}u_{ik}v_{jk}\right)}^{2}$$

where $a_{ij}$ denotes the element at the i-th row and the j-th column in dictionary A, R denotes the number of chosen principal components, and $V_{j:}$ denotes the j-th row of matrix V. One can find the details about NMF in [47]. For the admirable properties of the nonnegativity of NMF, Zhang et al. and Cai et al. proposed a topology-preserving nonnegative matrix factorization (TPNMF) method and a graph regularized nonnegative matrix factorization (GNMF) method, respectively [47,48]. 

Since the solution of NMF is not unique, Liu et al. and Zhang et al. proposed its surrogate, called nonnegative garrote (NNG), for nonnegative sparse representation [49,50]:

(5) $$\underset{x}{\min}{\left\|y-Ax\right\|}_{2}^{2}+\gamma \sum_{i=1}^{L}x_{i}\;s.t.\;x_{i}\geq 0$$

where Equation (5) can be solved by referring to [51]. Since NNG replaced the *l*_1_-norm with a summation term, so it relaxed the sparsity constraint regarding the coding x. 

Ji et al. proposed a genuine nonnegative sparse coding method by directly imposing a nonnegative constraint on sparse representation [52]. However, they adopted the numerical methods to solve that. Such a compromised solving approach is inefficient and imprecise.

### 2.3. Error Estimation

To well measure the coding errors, some novel fidelity terms are proposed to replace the *l*_1_- or *l*_2_-norm. CESR measured the similarity between the query sample y and its reconstruction Ax by utilizing the correntropy-induced metric [29]:

(6) $$\underset{x}{\max}\sum_{i=1}^{D}I\left(y_{i}-A_{i:}x\right)-\gamma \sum_{i=1}^{L}x_{i}\;s.t.\;x_{i}\geq 0$$

where $I\left(e_{i}\right)=\exp\left(-e_{i}^{2}/2\sigma^{2}\right)$ is a metric function, and σ is a kernel parameter. Meanwhile, $e_{i}=y_{i}-A_{i:}x$ is the i-th element of the error e, and $A_{i:}$ and $y_{i}$ denote the i-th row of dictionary A and the i-th element of the query sample y, respectively.

RRC assumed the elements in error ***e*** and coding ***x*** are i.i.d. with the probability densities functions (PDF) $m\left(e_{i}\right)$ and $n\left(x_{i}\right)$, respectively. Let $\ell \left(e_{i}\right)=-\ln m\left(e_{i}\right)$ and $\theta \left(x_{i}\right)=-\ln n\left(x_{i}\right)$. The local quadratic approximation of $\ell \left(e_{i}\right)$ produces a weighted function $w_{i}^{t}=\dot{\ell }\left(e_{i}^{t}\right)/e_{i}^{t}$ to minimize $\sum_{i=1}^{D}\ell \left(e_{i}\right)+\sum_{i=1}^{L}\theta \left(x_{i}\right)$ in an iteratively reweighted way, where $\dot{\ell }$ is the first-order derivation of function ℓ. Empirically, the Logistic function was selected as the weighted function [28]:

(7) $$w_{i}=\exp\left(-\mu e_{i}^{2}+\mu \delta \right)/\left(1+\exp\left(-\mu e_{i}^{2}+\mu \delta \right)\right)$$

where the parameters μ and δ control the decreasing rate and the demarcation point, respectively. Assuming coding x follows a Gaussian distribution [28], the minimization problem can be finally reduced to:

(8) $$\underset{x}{\min}{\left\|\sqrt{W}\left(y-Ax\right)\right\|}_{2}^{2}+\gamma {\left\|x\right\|}_{1}$$

where W=diag(W) is an error detector, and elements in vector w can be obtained according to Equation (7).

LUMIRC carried out a Laplacian-uniform mixture function $\ell \left(e_{i}\right)=\alpha \left(\exp\left(-\left|e_{i}\right|/b\right)+c\right)$ to fit the empirical errors [31]. The corresponding weighted function is obtained by:

(9) $$w_{i}=\dot{\ell }\left(e_{i}^{t}\right)=\exp\left(-\left|e_{i}\right|/b\right)/\left(\exp\left(-\left|e_{i}\right|/b\right)+c\right)$$

where b controls the decreasing rate, and c is a constant. Thus, LUMIRC can be reformulated by:

(10) $$\underset{x}{\min}{\left\|\sqrt{W}e\right\|}_{1}+\gamma {\left\|x\right\|}_{1}\;s.t.\;e=y-Ax$$

where w=diag(w), and elements in vector w can be obtained according to Equation (9).

It can be found that both RRC and LUMIRC chose the weighted function in a heuristic or empirical way, so their underlying ideas deserve in-depth analysis. The correntropy metric showing admirable properties on measuring coding errors was proved to be robust to the non-Gaussian noises and large outliers [40]. It also has the flexibility of adaptively adjusting fewer parameters compared with RRC and LUMIRC (see Equations (7), (9), and (21)). Due to these advantages, Lu et al. and Zhou et al. utilized the correntropy metric for robust subspace clustering and feature selection [35,48]. Wang et al. introduced it into the matching pursuit algorithm to propose a correntropy matching pursuit (CMP) method [34]. Unlike these work that achieved their goals with a simple introduction of correntropy metric, we equip the correntropy metric with a tailored regularizer to pursue stronger robustness.

## 3. Correntropy-Induced Discriminative Nonnegative Sparse Coding

In the coding process, CEE removes the contaminated pixels in the query sample, while DNSR extracts significant correct features for the subsequent classification. Accordingly, the framework of CDNSC is defined as follows:

(11) $$\underset{x}{\min}\sum_{i=1}^{D}\nu \left(e_{i}\right)+\sum_{i=1}^{L}\upsilon \left(x_{i}\right)$$

where $\nu \left(e_{i}\right)$ refers to CEE, and $\upsilon \left(x_{i}\right)$ refers to DNSR. We can obtain the specific formulation (Formula (27)) of CDNSC by substituting the formulized CEE (Formula (19)) and DNSR (Formula (26)) discussed in the subsequent Subsections into (11). For the specific implementation of CDNSC, one can refer to the operating steps listed in Algorithm 1, where the detailed calculations of all the involved variables are also given.

For this purpose, we introduce CDNSC from the following aspects: cooperative error estimator, discriminative nonnegative sparse regularizer, the optimization of CDNSC, and the extended CDNSC.

### 3.1. Cooperative Error Estimator

From the perspective of information learning [40], Liu et al. defined the correntropy between the query sample y and its reconstruction duplicate $y^{\prime }$ as:

(12) $$V_{\sigma }\left(y,y^{\prime }\right)=\iint I_{\sigma }\left(y-y^{\prime }\right)p_{yy^{\prime }}\left(y,y^{\prime }\right)dydy^{\prime }$$

where the joint PDF $p_{yy^{\prime }}\left(y,y^{\prime }\right)$ between y and $y^{\prime }$ is unknown in practice, which leads to a reduced estimator for the correntropy:

(13) $${\hat{V}}_{\sigma }\left(y,y^{\prime }\right)=\frac{1}{D}\sum_{i=1}^{D}I_{\sigma }\left(y_{i}-y_{i}^{\prime }\right).$$

Based on (13), the correntropy was extended into a general similarity metric between two arbitrary variables y and $y^{\prime }$, which is called the correntropy-induced metric (CIM):

(14) $${\mathrm{CIM}}_{\sigma }\left(y,y^{\prime }\right)={\left(I_{\sigma }\left(0\right)-\frac{1}{D}\sum_{i=1}^{D}I_{\sigma }\left(e_{i}\right)\right)}^{\frac{1}{2}}$$

where $e_{i}$ is the i-th element of the variable e, and $e=y-y^{\prime }$. Formula (14) has been verified to be a well-defined metric for satisfying the properties of nonnegativity, symmetry, etc. [53].

**Figure 2:**
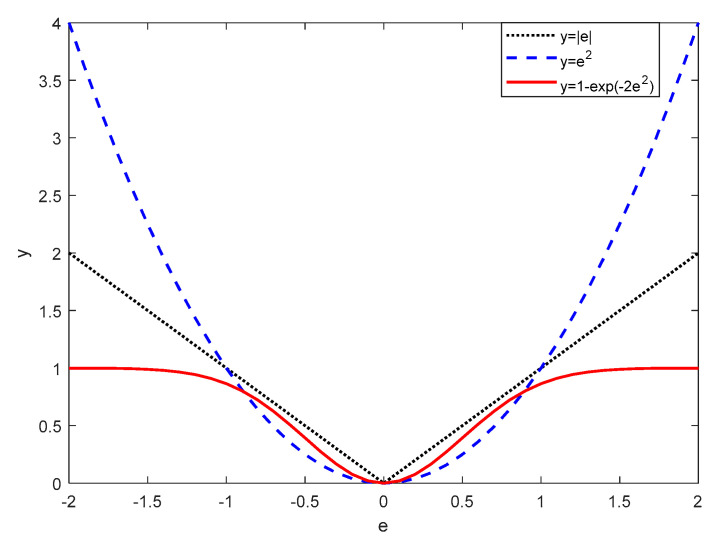
Comparison among the absolute error metric, MSE metric, and CIM.

Figure 2 shows the comparison among the absolute error metric, mean squared error (MSE) metric, and CIM. It is clear that the absolute error metric is a real expression of errors, while the squared error matric quadratically expresses errors. As global metrics, both of them are sensitive to large errors. Interestingly, the CIM is close to the absolute error metric and MSE metric when errors are small, and it tends to 1 when errors get larger. Note that large errors are usually caused by non-Gaussian corruption and continuous occlusion [54]. Hence, CIM is robust to them.

In the regression-based palmprint recognition procedures, we naturally hope that the representation of the query sample y can be unaffected by the contaminations, and y can be well reflected by the extracted features. Fortunately, the CIM can support us to find such a kind of representation by:

(15) $$\underset{x}{\min}{\mathrm{CIM}}_{\sigma }\left(y,Ax\right)=\underset{x}{\min}\frac{1}{D}\sum_{i=1}^{D}\left(1-I_{\sigma }\left(e_{i}\right)\right)\;s.t.\;y-Ax=e.$$

Although the gradient descent algorithm can be utilized to solve (15), we prefer to leverage the HQ method as it’s more effective and can provide an adaptive weighted variable for error detection. To well solve problem (15), Proposition 1 is introduced as follows (the proof of proposition 1 is provided in Appendix A).

**Proposition** **1.** *For (15), there exists a dual function* ψ *such that:*

(16) $$1-I_{\sigma }\left(e_{i}\right)=\underset{w_{i}\in \mathbb{R}}{\inf}\left\{\frac{1}{2}w_{i}e_{i}^{2}+\psi \left(w_{i}\right)\right\},$$

*and its minimum is reached at:*

(17) $$w_{i}=\frac{1}{\sigma^{2}}\exp\left(-e_{i}^{2}/2\sigma^{2}\right).$$

Equation (17) indicates that the CIM can adaptively learn small weights to suppress the large errors and assign significant weights to the relatively pure pixels to manifest their importance. Compared with RRC and LUMIRC, it’s easier to perform CIM towards various contaminations by adaptively adjusting the parameter σ:

(18) $$\sigma^{2}=\frac{1}{2D}{\left\|y-Ax\right\|}_{2}^{2}.$$

Assuming the undetected contaminations are sparse, based on (15) and Proposition 1, CEE can be formulized as:

(19) $$\sum_{i=1}^{D}\nu \left(e_{i}\right)={\left\|\sqrt{W}e\right\|}_{1}\;s.t.\;y-Ax=e$$

where W=diag(w) is an error detector, and elements in vector w can be obtained according to (17). Meanwhile, the *l*_1_-norm is an error corrector.

### 3.2. Discriminative Nonnegative Sparse Regularizer

As an important part of DNSR, the discriminative constraint term is designed as:

(20) $$\sum_{i=1}^{L}\upsilon_{1}\left(x_{i}\right)=\sum_{i=1}^{L}\sum_{j=1}^{L}{\left(A_{:i}x_{i}\right)}^{T}W\left(A_{:j}x_{j}\right)$$

where the superscript T denotes the matrix transpose. The minimization of (20) means the representation of the i-th and the j-th classes has the lowest correlation, which enables the representation of diverse classes to be discriminative. Thus, the method prefers to select the most relevant samples to represent the query sample. This encourages the coefficients to be intrinsically sparse. Note matrix W is obtained by (17), which suppresses errors from affecting the discriminative coding. Hence, minimizing (20) encourages x to be robustly sparse.

In light of the drawbacks of NMF and NNG, we directly impose a nonnegative constraint on the sparse representation:

(21) $$\sum_{i=1}^{L}\upsilon_{2}\left(x_{i}\right)=\sum_{i=1}^{L}\left|x_{i}\right|\;s.t.\;x_{i}\geq 0.$$

Different from [50], we aim to develop an efficient solving method to explore the analytical solution of (21). To the best of our knowledge, there has no method can be directly exploited. Fortunately, we can refer to the Lagrange multiplier theorem to convert the inequality constraint problem (ICP) (21) into an equality constraint problem (ECP). Now, we consider a general ICP:

(22) $$\underset{t}{\min}f\left(t\right)\;s.t.\;g\left(t\right)\geq 0$$

where $t\in {\mathbb{R}}^{1}$. Then, the corresponding ECP of (22) reads:

(23) $$\underset{t}{\min}f\left(t\right)\;s.t.\;h\left(t,z\right)=g\left(t\right)-z^{2}=0$$

where z is an auxiliary variable to describe the nonnegativity of the value of function g(t). We manage to prove that (23) has the same Karush-Kuhn-Tucker (KKT) conditions as (22), which promises that (23) is an equivalent transformation of (22) under the Lagrange multiplier theorem-based optimization method. Consequently, Lemma 1 is introduced as follows (the proof of lemma 1 is provided in Appendix B).

**Lemma 1.** **1.** *Assuming* $t^{*}$ *is a local minimum of (22), and* f(t) *and* g(t) *are continuously differentiable, there exists a unique* $\varphi^{*}$ *for (23) such that:*

(24) $$\{\begin{array}{c} \nabla_{t}L\left(t^{*},z^{*},\varphi^{*}\right)=\nabla_{t}f\left(t^{*}\right)+\varphi^{*}\nabla_{t}h\left(t^{*},z^{*}\right)=0\\ \varphi^{*}\leq 0. \end{array}$$

*where* ∇ *denotes the first-order differential operator.*

Because (23) and (22) have the same KKT conditions (refer to proposition 3.3.1 in [55] to find the KKT conditions of (22)), we conclude that solving (23) is equivalent to solve (22) under the Lagrange multiplier method. So, (21) can be rewritten as:

(25) $$\sum_{i=1}^{L}{\bar{\upsilon }}_{2}\left(x_{i}\right)=\sum_{i=1}^{L}\left|x_{i}\right|\;s.t.\;x_{i}=z_{i}^{2}.$$

Combining (20) and (25), DNSR can be formulized as:

(26) $$\sum_{i=1}^{L}\upsilon \left(x_{i}\right)=\sum_{i=1}^{L}\sum_{j=1}^{L}{\left(A_{:i}x_{i}\right)}^{T}W\left(A_{:j}x_{j}\right)+\sum_{i=1}^{L}\left|x_{i}\right|\;s.t.\;x_{i}=z_{i}^{2}.$$

### 3.3. Optimization of CDNSC

We obtain the unified CDNSC by substituting (21) and (26) into (11):

(27) $$\begin{array}{l} J\left(W,e,x,z\right)=\underset{W,e,x,z}{\min}{\left\|\sqrt{W}e\right\|}_{1}+\alpha \sum_{i=1}^{L}\sum_{j=1}^{L}{\left(A_{:i}x_{i}\right)}^{T}W\left(A_{:j}x_{j}\right)+\beta {\left\|x\right\|}_{1}\\ s.t.\;\;\begin{array}{c} y-Ax=e,x=z^{2} \end{array} \end{array}$$

where α and β are two tunable parameters, and the vector $z^{2}$ is composed of the element $z_{i}^{2}$, i=1,⋯,L. Note (27) can be rewritten as:

(28) $$\begin{array}{l} J\left(\tilde{e},u,x,z\right)=\underset{\tilde{e},u,x,z}{\min}{\left\|\tilde{e}\right\|}_{1}+\alpha \sum_{i=1}^{L}\sum_{j=1}^{L}{\left({\tilde{A}}_{:i}x_{i}\right)}^{T}\left({\tilde{A}}_{:j}x_{j}\right)+\beta {\left\|u\right\|}_{1}\\ s.t.\;\;{\begin{array}{c} \tilde{y}-\tilde{A}x=\tilde{e},x=z \end{array}}^{2},x=u \end{array}$$

where $\tilde{e}=\sqrt{W}e$, $\tilde{y}=\sqrt{W}y$, and $\tilde{A}=\sqrt{W}A$. Let $\phi_{1}$, $\phi_{2}$, and $\phi_{3}$ be three vectors of the Lagrange multipliers, and ρ be the penalty parameter, the augmented Lagrange function of (28) reads:

(29) $$\begin{array}{l} ℒ\left(\tilde{e},u,x,z,\phi_{1},\phi_{2},\phi_{3},\rho \right)={\left\|\tilde{e}\right\|}_{1}+\alpha \sum_{i=1}^{L}\sum_{j=1}^{L}{\left({\tilde{A}}_{:i}x_{i}\right)}^{T}\left({\tilde{A}}_{:j}x_{j}\right)\\ +\beta {\left\|u\right\|}_{1}+\phi_{1}^{T}\left(\tilde{y}-\tilde{A}x-\tilde{e}\right)+\phi_{2}^{T}\left(x-z^{2}\right)+\phi_{3}^{T}\left(x-u\right)+\\ \frac{\rho }{2}\left({\left\|\tilde{y}-\tilde{A}x-\tilde{e}\right\|}_{2}^{2}+{\left\|x-z^{2}\right\|}_{2}^{2}+{\left\|x-u\right\|}_{2}^{2}\right). \end{array}$$

Before solving (29), the introduced auxiliary variable z should be eliminated. Let $\partial ℒ\left(\tilde{e},u,x,z,\phi_{1},\phi_{2},\phi_{3},\rho \right)/\partial z_{i}=0$, we have:

(30) $$x_{i}-z_{i}^{2}=\{\begin{array}{c} \begin{array}{cc} -\phi_{2,i}/\rho , & \rho x_{i}+\phi_{2,i}>0 \end{array}\\ \begin{array}{cc} x_{i}, & \rho x_{i}+\phi_{2,i}\leq 0 \end{array} \end{array}$$

where $\phi_{2,i}$ is the i-th element of the Lagrange multiplier $\phi_{2}$. Note the selection function (30) renders (29) nondifferentiable. To obtain the analytical solution of (29), we skillfully rewrite (30) by:

(31) $$\phi_{2,i}+\rho \left(x_{i}-z_{i}^{2}\right)=\sqrt{b_{i}}\left(\rho x_{i}+\phi_{2,i}\right)$$

where the element $b_{i}$ is determined by:

(32) $$b_{i}=\{\begin{array}{c} \begin{array}{cc} 0, & \rho x_{i}+\phi_{2,i}>0 \end{array}\\ \begin{array}{cc} 1, & \rho x_{i}+\phi_{2,i}\leq 0. \end{array} \end{array}$$

Accordingly, problem (29) can be further rewritten as:

(33) $$\begin{array}{l} \bar{ℒ}\left(\tilde{e},B,u,x,\phi_{1},\phi_{2},\phi_{3},\rho \right)={\left\|\tilde{e}\right\|}_{1}+\alpha \sum_{i=1}^{L}\sum_{j=1}^{L}{\left({\tilde{A}}_{:i}x_{i}\right)}^{T}\left({\tilde{A}}_{:j}x_{j}\right)+\beta {\left\|u\right\|}_{1}\\ \frac{\rho }{2}{\left\|\tilde{e}-\left(\tilde{y}-\tilde{A}x+\frac{\phi_{1}}{\rho }\right)\right\|}_{2}^{2}+\frac{1}{2\rho }{\left\|\sqrt{B}\left(\rho x+\phi_{2}\right)\right\|}_{2}^{2}+\frac{\rho }{2}{\left\|u-\left(x+\frac{\phi_{3}}{\rho }\right)\right\|}_{2}^{2} \end{array}$$

where B=diag(b), and the element $b_{i}$ in vector b is determined by (32).

In the l-th iteration, once matrix $W^{l+1}$ is updated by (17) and fixed, the variables $\tilde{y}$ and $\tilde{A}$ are also fixed. ADMM [56] respectively updates each undetermined variable in (33) as follows:

(34) $${\tilde{e}}^{l+1}=\underset{\tilde{e}}{\arg\min}ℒ\left(\tilde{e},x^{l},\phi_{1}^{l},\rho \right)$$

(35) $$B^{l+1}=\underset{B}{\arg\min}ℒ\left(x^{l},\phi_{2}^{l},\rho \right)$$

(36) $$u^{l+1}=\underset{u}{\arg\min}ℒ\left(u,x^{l},\phi_{3}^{l},\rho \right)$$

(37) $$x^{l+1}=\underset{x}{\arg\min}ℒ\left({\tilde{e}}^{l+1},B^{l+1},u^{l+1},x,\phi_{1}^{l},\phi_{2}^{l},\phi_{3}^{l},\rho \right)$$

(38) $$\phi_{1}^{l+1}=\phi_{1}^{l}+\rho^{l}\left(\tilde{y}-\tilde{A}x^{l+1}-{\tilde{e}}^{l+1}\right)$$

(39) $$\phi_{2}^{l+1}=\sqrt{B^{l+1}}\left(\rho^{l}x^{l+1}+\phi_{2}^{l}\right)$$

(40) $$\phi_{3}^{l+1}=\phi_{3}^{l}+\rho^{l}\left(x^{l+1}-u^{l+1}\right)$$

(41) $$\rho^{l+1}=\min\left(\mu \rho^{l},\rho_{\max}\right)$$

where the parameter μ>1, and (39) is obtained by substituting (31) into the formula $\phi_{2}^{l+1}=\phi_{2}^{l}+\rho^{l}\left(x^{l+1}-{\left(z^{l+1}\right)}^{2}\right)$. Note (39) reveals that the Lagrange multiplier $\phi_{2}^{l+1}\leq 0$ always holds, which is consistent with the Lemma 1. For (34), we have:

(42) $${\tilde{e}}^{l+1}=\underset{\tilde{e}}{\arg\min}{\left\|\tilde{e}\right\|}_{1}+\frac{\rho^{l}}{2}{\left\|\tilde{e}-d_{1}^{l}\right\|}_{2}^{2}$$

where the variable $d_{1}^{l}=\tilde{y}-\tilde{A}x^{l}+\phi_{1}{}^{l}/\rho^{l}$. The subproblem (42) can be explicitly solved by the soft thresholding function:

(43) $${\tilde{e}}^{l+1}=\mathrm{sign}\left(d_{1}^{l}\right)\max\left\{\left|d_{1}^{l}\right|-1/\rho^{l},0\right\}.$$

The variable $B^{l+1}$ in the subproblem (35) is updated by formula (32), and the subproblem (36) can be expressed as:

(44) $$u^{l+1}=\underset{u}{\arg\min}{\left\|u\right\|}_{1}+\frac{\rho^{l}}{2}{\left\|u-d_{2}^{l}\right\|}_{2}^{2}$$

where the variable $d_{2}^{l}=x^{l}+\phi_{3}{}^{l}/\rho^{l}$. Similar to (42), (44) is solved by:

(45) $$u^{l+1}=\mathrm{sign}\left(d_{2}^{l}\right)\max\left\{\left|d_{2}^{l}\right|-\beta /\rho^{l},0\right\}.$$

For the subproblem (45), we have:

(46) $$\begin{array}{l} x^{l+1}=\underset{x}{\arg\min}\alpha \sum_{i=1}^{L}\sum_{j=1}^{L}{\left({\tilde{A}}_{:i}x_{i}\right)}^{T}\left({\tilde{A}}_{:j}x_{j}\right)+\frac{\rho^{l}}{2}{\left\|{\tilde{e}}^{l}-\left(\tilde{y}-\tilde{A}x+\frac{\phi_{1}^{l}}{\rho^{l}}\right)\right\|}_{2}^{2}\\ \;\;\;\;\;\;\;\;\;\;\;\;\;\;\;\;\;\;\;+\frac{1}{2\rho^{l}}{\left\|\sqrt{B^{l}}\left(\rho^{l}x+\phi_{2}^{l}\right)\right\|}_{2}^{2}+\frac{\rho^{l}}{2}{\left\|u^{l}-\left(x+\frac{\phi_{3}^{l}}{\rho^{l}}\right)\right\|}_{2}^{2}. \end{array}$$

Before solving problem (46), we specifically consider the derivative of the discriminative term over the variable $x_{n}$:

(47) $$\begin{array}{l} \;\;\frac{\partial }{\partial x_{n}}\left[\sum_{i=1}^{L}\sum_{j=1}^{L}{\left({\tilde{A}}_{:i}x_{i}\right)}^{T}\left({\tilde{A}}_{:j}x_{j}\right)\right]\\ =\frac{\partial }{\partial x_{n}}[\sum_{\begin{array}{l} i=1\\ i\neq n \end{array}}^{L}{\left({\tilde{A}}_{:i}x_{i}\right)}^{T}\left({\tilde{A}}_{:j}x_{j}\right)+\sum_{\begin{array}{l} j=1\\ j\neq n \end{array}}^{L}{\left({\tilde{A}}_{:i}x_{i}\right)}^{T}\left({\tilde{A}}_{:j}x_{j}\right)\\ \;\;\;\;\;\;\;\;+\sum_{\begin{array}{l} i=1\\ i\neq n \end{array}}^{L}\sum_{\begin{array}{l} j=1\\ j\neq n \end{array}}^{L}{\left({\tilde{A}}_{:i}x_{i}\right)}^{T}\left({\tilde{A}}_{:j}x_{j}\right)+{\left({\tilde{A}}_{:i}x_{i}\right)}^{T}\left({\tilde{A}}_{:j}x_{j}\right)]\\ =2\left[\sum_{\begin{array}{l} i=1\\ i\neq n \end{array}}^{L}{\tilde{A}}_{:n}^{T}\left({\tilde{A}}_{:i}x_{i}\right)+{\tilde{A}}_{:n}^{T}\left({\tilde{A}}_{:i}x_{i}\right)\right]\\ =2{\tilde{A}}_{:n}^{T}\tilde{A}x. \end{array}$$

Accordingly, we have $\partial \left(\sum_{i=1}^{L}\sum_{j=1}^{L}{\left({\tilde{A}}_{:i}x_{i}\right)}^{T}\left({\tilde{A}}_{:j}x_{j}\right)\right)/\partial x=2{\tilde{A}}^{T}\tilde{A}x$. Hence, a closed-form solution of (46) is obtained:

(48) $$\begin{array}{l} x^{l+1}={\left(2\alpha {\tilde{A}}^{T}\tilde{A}+\rho^{l}{\tilde{A}}^{T}\tilde{A}+\rho^{l}B^{l+1}+\rho^{l}\right)}^{-1}\\ (\rho^{l}u^{l+1}-\phi_{3}^{l}-B^{l+1}\phi_{2}^{l}-\rho^{l}{\tilde{A}}^{T}\left({\tilde{e}}^{l+1}-\tilde{y}-\phi_{1}^{l}/\rho^{l}\right)). \end{array}$$

The total optimization procedures of CDNSC are summarized in Algorithm 1. A termination criterion is enforced to verify whether Algorithm 1 converges

(49) $${\left\|x^{l+1}-x^{l}\right\|}_{2}/{\left\|x^{l}\right\|}_{2}<\varepsilon$$

where ε>0 is a small stopping value.
**Algorithm 1.** Optimization of CDNSC via ADMM**Input:**$A,y,\alpha ,\beta ,\mu ,\rho_{\max},k_{\max}$, and ε.**Output:** The optimal ${\tilde{y}}^{*}$, ${\tilde{A}}^{*}$, ${\tilde{e}}^{*}$, and $x^{*}$.**Initialization:**k=0, $x^{k}=1/L$.**Repeat**1: k=k+1;2: Estimate weight matrix $W^{k}$ by (17) and (20);
**Update:**$\tilde{A}=\sqrt{W^{k}}A$ and $\tilde{y}=\sqrt{W^{k}}y$.
** Initialization:**l=0, $x^{l}=x^{k}$, $\phi_{1}^{l}$, $\phi_{2}^{l}$, $\phi_{3}^{l}$, and $\rho^{l}$. **Repeat** 3: l=l+1;
 4: Estimate ${\tilde{e}}^{l}$ by (43); 5: Update $B^{l}$ by (32);  6: Estimate $u^{l}$ by (45); 7: Estimate ${\tilde{x}}^{l}$ by (48); 8: Update $\phi_{1}^{l}$, $\phi_{2}^{l}$, $\phi_{3}^{l}$, and $\rho^{l}$ by (38), (39), (40), and (41); 9: Check the termination criterion by (49); **Until convergence**11: $x^{k}=x^{l}$
**Until**$k>k_{\max}$

We classify y by finding the least reconstruction error holder among all classes. Therefore, the CDNSC-driven classifier is formulized as follows:

(50) $$\mathrm{ID}=\underset{c}{\arg\min}{\left\|{\tilde{y}}^{*}-{\tilde{A}}^{*}\delta_{c}\left(x^{*}\right)-{\tilde{e}}^{*}\right\|}_{2}$$

where the superscript ∗ indicates the convergence values, and the function $\delta_{c}$ selects the entries affiliated to the c-th class.

### 3.4. Extended CDNSC

Before presenting the extended CDNSC (E-CDNSC), we first establish the objective function to learn the feasible parameters to fuse the features of different spectrums. Let $\lambda_{s}$ be the fusion parameters corresponding to the features of the s-th spectrum, the E-CDNSC-driven classifier is given by:

(51) $$\mathrm{ID}\left(\lambda \right)=\underset{c}{\arg\min}{\sum_{s=1}^{S}\lambda_{s}\left\|{\tilde{y}}_{s}^{*}-{\tilde{A}}_{s}^{*}\delta_{c}\left(x_{s}^{*}\right)-{\tilde{e}}_{s}^{*}\right\|}_{2}$$

where the variables ${\tilde{y}}_{s}^{*}$,${\tilde{A}}_{s}^{*}$, and ${\tilde{e}}_{s}^{*}$ are the output of Algorithm 1. Since the parameter $\lambda_{s}$ should be nonnegative, and its summation should be equal to 1, we impose two constraints to define the feasible region of the vector λ and deem the recognition rate as the objective to establish the objective function regarding λ:

(52) $$\underset{\lambda }{\max}R\left(\lambda \right)=\underset{\lambda }{\max}\frac{\sum_{i=1}^{N}\Theta_{i}\left(\mathrm{ID}\left(\lambda \right)\right)}{N}\times 100\;\;s.t.\lambda_{s}\geq 0,\;\sum_{s=1}^{S}\lambda_{s}=1$$

where the variable N denotes the number of the test samples, and the function Θ(ID(λ)) counts the correctly recognized samples.

For Equation (52) is nondifferentiable, we propose a modified intelligent optimizer, named constrained PSO (CPSO), to solve it. Note the first constraint can be addressed by setting a nonnegative flying region for the particle swarm. Then, inspired by the Lagrange method, the second constraint is addressed by:

(53) $$\underset{\lambda }{\min}\;R\;˜\left(\lambda \right)=\underset{\lambda }{\min}\left(-R\left(\lambda \right)+\eta {\left\|\sum_{s=1}^{S}\lambda_{s}-1\right\|}_{2}\right)\times 100$$

where η>0 is a penalty parameter.

In the optimizing process, CPSO ceaselessly produces the particle swarm $P^{m}\in {\mathbb{R}}_{}^{Q\times S}$ to randomly fly in the defined region, where the variable $P^{m}$ denotes the particle swarm in the m-th generation, and the variables Q and S denote the individual number and particle swarm dimension, respectively. Note each row of $P^{m}$ signifies a potential solution to minimize (53). Specifically, CPSO finds the best individual $p^{*}$ from $P^{1}$ to minimize (53) in the first generation, then $p^{*}$ reproduces $P^{2}$ in the second generation. The above processes repeat until the following termination criterion is met:

(54) $${\left\|\sum_{s=1}^{S}\lambda_{s}^{m+1}-1\right\|}_{2}<\xi$$

where ξ is a small positive value. Referring to 57, we update the penalty parameter in each generation by:

(55) $$\eta^{m+1}=\min\left(\varsigma \eta^{m},\eta_{\max}\right)$$

where $\eta_{\max}$ is a large positive value, and ς is a small positive value. The procedures of optimizing (53) is outlined in Algorithm 2 (In each generation, CPSO reproduces new particle swarm in the same way as PSO. For the limited space, we omit that here. The details can be found in [57]).
**Algorithm 2.** Optimization of (53) via CPSO**Input:**${\tilde{y}}_{s}^{*},{\tilde{A}}_{s}^{*},x_{s}^{*},{\tilde{e}}_{s}^{*},\eta_{0},\varsigma ,\eta_{\max},\xi ,Q$, and S**Output:** The optimal $\lambda^{*}$**Initialization:**m=0, particle swarm $P^{m}$**Repeat** 1: Calculate the fitness value of each individual in $P^{m}$ on (53);
 2: Find the individual $p^{*}$ in $P^{m}$ with least fitness value; 3: $\lambda =p^{*}$; 4: m=m+1; 5: Reproduce particle swarm $P^{m}$ around $p^{*}$; 6: Update $\eta^{m}$ by (55); 7: Check the termination criterion by (54);**Until convergence**

## 4. Analysis of CDNSC

This section discusses the effectiveness of CDNSC by analyzing its complexity and convergence and demonstrating the positive effect of DNSR on the performance of CEE.

### 4.1. Complexity and Convergence of CDNSC

Although the mathematical derivation of optimizing CDNSC seems to be complicated due to the nonnegative constraint, the resulted extra computation is only to construct a simple matrix B, which has a low computation complexity of O(L). The subproblems regarding the parameters $\tilde{e}$ and $\tilde{u}$ can be explicitly solved by the simple soft thresholding method, so the computational complexity of solving $\tilde{e}$ and u is O(L). When solving the parameter $\tilde{x}$, the most time-consuming process is the matrix inversion, which has a complexity of $O\left(L^{2}\right)$. Let k and l signify the iteration index of the outer loop and inner loop in Algorithm 1, respectively. Ignoring the basic operation like matrix addition and subtraction, the computational complexity of algorithm 1 is $O\left(kl\left(3L+L^{2}\right)\right)$. Unlike the Lasso problem that should be solved iteratively, all the *l*_1_ minimization problems in CDNSC have closed-form solutions, so CDNSC is relatively efficient.

The convergence of CDNSC is illustrated in Proposition 2 (the rough proof of proposition 2 is provided in Appendix C).

**Proposition** **2.** *The sequence* $\bar{ℒ}\left(W^{k},x^{k},e^{l},B^{l},u^{l},\phi_{1}{}^{l},\phi_{2}{}^{l},\phi_{3}{}^{l},\rho^{l}\right)$ *generated by Algorithm 1 converges.*

### 4.2. Positive Effect of DNSR to CEE

**Figure 3:**
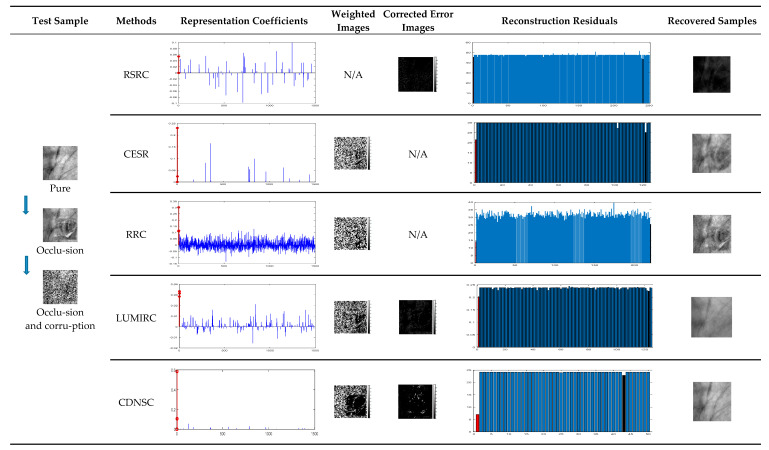
Comprehensive comparisons between CDNSC and the state-of-the-art methods.

To intuitively illustrate the positive effect of DNSR to CEE, the state-of-the-art methods on error correction or detection are selected for comparison. The experiments are performed on the blue spectrum samples in the PolyU palmprint database (all the samples are resized to 80 × 80 pixels and vectorially normalized). The first three samples of each subject are used for training, and a randomly selected sample of the first subject is chosen for test. We consider robust palmprint recognition under the mixed-contaminations and simulate it by imposing a combination of 40% block-wise scar occlusion and 40% pixel-wise corruption on the query sample. Figure 3 displays the performance of all the competing methods, where the coefficients and reconstruction residuals corresponding to the congeneric samples of the query sample are marked in red, while the reconstruction residual closest to the congeneric reconstruction residual are marked in black (‘N/A’ indicates that the corresponding method lacks for error corrector or detector).

Without an error detector, RSRC can only correct a portion of corruption, which leads to the misclassification and a terrible recovery of the query image. By contrast, CESR and RRC lack for the error corrector. This puts great pressure on their error detectors, so the undetected errors affect the sparse coding and result in indistinguishable inter-class reconstruction residuals, which can be possible to mislead the classifier. Since LUMIRC neglects to learn a proper regularizer, it’s representation coefficients are not sparse enough, and its error estimator appears to be underpowered. Benefitting from DNSR that encourages the sparsity and nonnegativity of the coefficients, CDNSC presents sparser coefficients than the other competitors, in which elements corresponding to the real class are significantly large and physically meaningful. So, CEE presents more precise error estimation results, and the inter-class reconstruction residuals are more distinguishable for the classification.

## 5. Experiments

This section verifies the flexibility and robustness of CDNSC concerning various contaminations. Meanwhile, to facilitate the intuitive comparison of the recognition accuracy between the single-spectrum and multispectral palmprint recognition, we choose the two public multispectral palmprint databases, CASIA database and PolyU database, as the benchmarks.

### 5.1. Experimental Settings

#### 5.1.1. CASIA Database 

**Figure 4:**
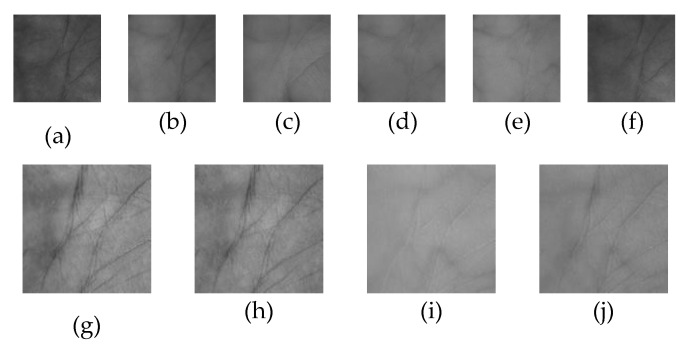
Some typical multispectral palmprint images in the PolyU database and CASIA database. (**a**–**f**) Samples under the 460 nm, 630 nm, 700 nm, 850 nm, 940 nm, and white spectrums in the CASIA database, (**g**–**j**) Samples under the Blue, Green, NIR, and Red spectrums in the PolyU database.

This database [58] was built by using a contactless device to capture palmprints. There are no pegs to restrict hand posture and position, so the variation of illumination and palm posture extensively exist in samples. Images of 200 palms were collected in two sessions with an interval of more than one month. In a session, each palm was captured three times, respectively under 460 nm, 630 nm, 700 nm, 850 nm, 940 nm, and white spectrua. There were six images acquired from one palm. The samples are all uncropped original palm images. We utilize the method in [1] to crop each sample with a size of 180 × 180 to obtain the ROI images. In the experiments, samples of each subject are randomly divided with the proportion of 3:1:2 to compose a dictionary set, a feature fusion training set, and a test set, respectively. Figure 4a–f show some typical multispectral samples in the CASIA database.

#### 5.1.2. PolyU Database

Samples in this database were captured by a contact-based device, where pegs are set to restrict hand posture and position. Hence, the acquired samples are rather regular. Palmprint images of 500 palms were collected in two sessions with an interval of nine days. In a session, each palm was captured six times, respectively under red, blue, green, and NIR spectra, so there were 12 images acquired from one palm. The ROI images were already cropped with a size of 128 × 128 by using the method in [59]. In the experiments, samples of each subject are randomly divided with the proportion of 1:1:1 to compose a dictionary set, a feature fusion training set, and a test set, respectively. Figure 4g–j show some typical multispectral palmprints in the PolyU database.

#### 5.1.3. Compared Methods

There are few classical methods proposed for robust palmprint recognition. Since CDNSC derives from concluding the merits and demerits of the robust regression-based methods, the optional competing methods are all based on robust regression analysis. To present convictive comparisons, the state-of-the-art methods on coding regularization, nonnegative representation, error correction, and error detection are all preferred. Specifically, LRC and the regularization-based SRC and CRC are selected. For the methods of nonnegative coding, the classical NNG and GNMF [60] are picked. As dictionary learning-based methods, DSRC and SSRC are chosen. Meanwhile, the state-of-the-art error correction and detection-based methods, including RSRC, CESR [61], *l*_1_-regularized RRC [61], and LUMIRC [61], are chosen. Finally, as a successful application of the correntropy, CMP is selected.

#### 5.1.4. Parameter Settings and Experimental Platform

Parameters in Algorithm 1 are set as α=β={0.01,0.1,1,10,50}, $\phi_{1}^{l}=\phi_{2}^{l}=\phi_{3}^{l}=0.1$, $\rho_{0}=1$, μ=1.5, $\rho_{\max}=1e+8$, $k_{\max}=3$, and ε=1e−3. On the basis, the other parameters in Algorithm 2 are set as $\eta_{0}=0.01$, ς=1.2, $\eta_{\max}=100$, and ξ=1e−4. All the experiments are performed in MATLAB R2019a on a laptop with 2.6-GHz CPU and 4-GB RAM.

### 5.2. Robust Contactless Palmprint Recognition

**Figure 5:**
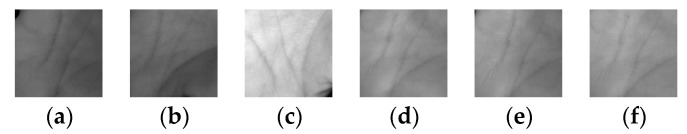
Variation of illumination and palm posture in the CASIA database. (**a**–**f**) respectively show the variation of illumination and palm posture among the palmprint ROI images.

Experiments in this part are all implemented on the 460 nm spectrum samples in the CASIA database. Without setting pegs to restrict hand posture and position, variation of illumination and palm posture exists extensively as shown in Figure 5, which brings some challenges to ROI segmentation and palmprint recognition. What’s more, in real-world applications, dense corruption and gross occlusion probably emerge in the query samples. Hence, we verify the robustness of CDNSC from the following aspects.

#### 5.2.1. Dimension and Number of Training Samples

**Figure 6:**
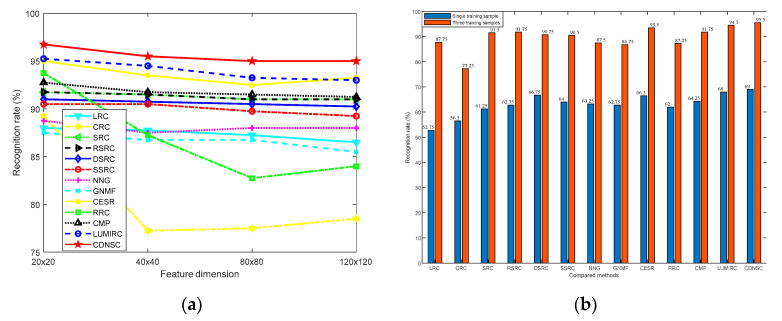
Recognition rate versus the dimension and number of training samples in the CASIA database. (**a**) Sample dimension, (**b**) Sample number.

As we know, the dimension and number of training samples often affect the performance of the biometric recognition methods. Here, we first consider the impact of sample dimension by fixing the training sample number of each subject as 3, where each sample is downsampled to the size of 20 × 20, 40 × 40, 80 × 80, and 120 × 120, respectively. When considering the impact of training sample number, we fix the sample dimension as 40 × 40 and respectively select the first sample and the first three samples of each subject in the dictionary set to compose the dictionary ***A***. The recognition rates of all the methods under the two cases are displayed in Figure 6a,b, respectively.

From Figure 6a, although both CDNSC and CESR adopt the correntropy metric as the error detector, CDNSC is more robust than CESR, which owns to the regularizer DNSR. As a strong competitor, RRC is more sensitive than CDNSC concerning the variation of sample dimension. It can be observed that CDNSC outperforms all the compared methods in each dimension case. When the sample dimension increases, the recognition rates of most compared methods present a slight downward trend. This is because a proper downsampling ratio contributes to getting rid of redundant pixels and extracting distinct features. Figure 6b indicates that CDNSC achieves better results than the others no matter with one or three training samples per class. Note we set rigorous parameters to solve all the Lasso problems to pursue the sparsity of coefficients, so the augmented dictionary in RSRC plays a little role to enhance the robustness of SRC.

#### 5.2.2. Continuous Scar Occlusion

**Figure 7:**
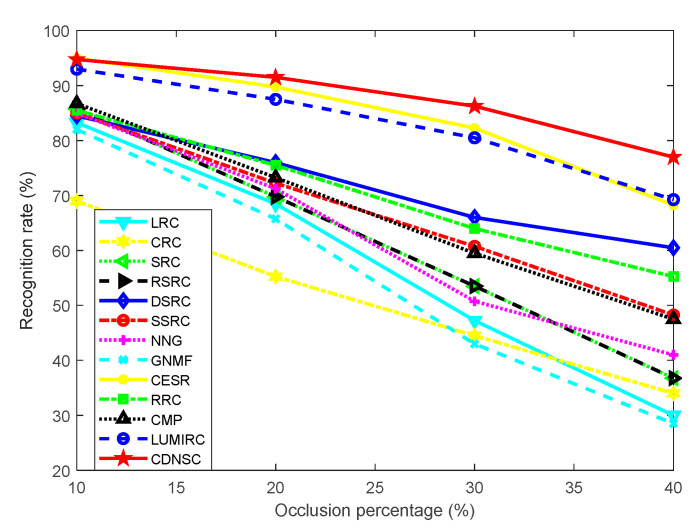
Recognition rate versus the level of scar occlusion.

We consider the possible occlusion caused by palm scar and design an experiment to investigate the robustness of CDNSC in handling the scar occlusion, a kind of continuous contamination. The sample dimension is fixed as 40 × 40, and the first three samples of each subject in the dictionary set of the CASIA database are all recruited to compose the dictionary ***A***. When performing the experiments, we randomly impose a scar image on the query samples to simulate the real scar. The percentage of scar occlusion varies from 10% to 40%. The experimental results are shown in Figure 7.

It’s evident that CDNSC outperforms other methods except CESR, at different occlusion levels. However, CDNSC seems to be less sensitive to the variation of occlusion level than CESR. Although CMP also adopts the correntropy metric, its performance is greatly degraded due to the continuous occlusion in comparison to its considerable performance on the original database (see Figure 6a).

#### 5.2.3. Dense Corruption and the Mixed-Contaminations

**Figure 8:**
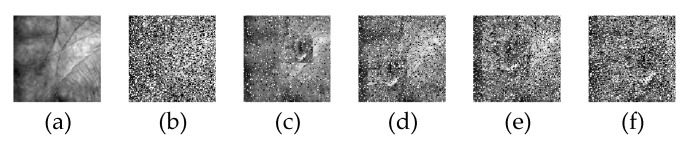
Query samples with dense corruption or mixed-contaminations in the test set of the CASIA database. (**a**) The original sample, (**b**) Sample with 50% corruption, (**c**–**f**) Samples with 10%–40% mixed-contaminations.

Finally, we consider the residual cases: dense corruption and the mixture of corruption and scar occlusion. The sample dimension and assembling processes of the dictionary ***A*** are similar to the above experiment. Due to CDNSC is quite robust to corruption, we directly evaluate its robustness regarding the dense corruption at the level of 50%. Besides, scar occlusion and corruption are combined to simulate the mixture case (level varies from 10% to 40%). The two kinds of contaminations are exhibited in Figure 8. Table 1 displays the experimental results of all the methods, where the best recognition rate of each case is bold.

Table 1 manifests that CDNSC and LUMIRC are particularly robust against dense corruption due to their appendant error correctors. But CDNSC achieves a higher recognition rate of 94.75% that is ahead of LUMIRC with 4.75%. Facing the mixed-contaminations, the compared methods seem to be fragile for the extra added corruption and present a great degeneration when the level of the mixed-contaminations increases, compared with their performance regarding occlusion (see Figure 7). Because DNSR makes CEE to be powerful, CDNSC is less sensitive to the increasing mixed-contaminations. This indicates that the proposed joint scheme is more flexible and robust to various challenging cases.

### 5.3. Robust Contact-Based Palmprint Recognition

Experiments in this part are all implemented on the blue spectrum samples in the PolyU database. Benefited from the well-defined acquisition restriction, samples in the PolyU database are quite regular. The recognition rate of CDNSC can reach to 100% on that. So we won’t make experiments on the original database anymore and directly verify the flexibility and robustness of CDNSC from the following aspects.

#### 5.3.1. Continuous Camera Lens Occlusion

**Figure 9:**
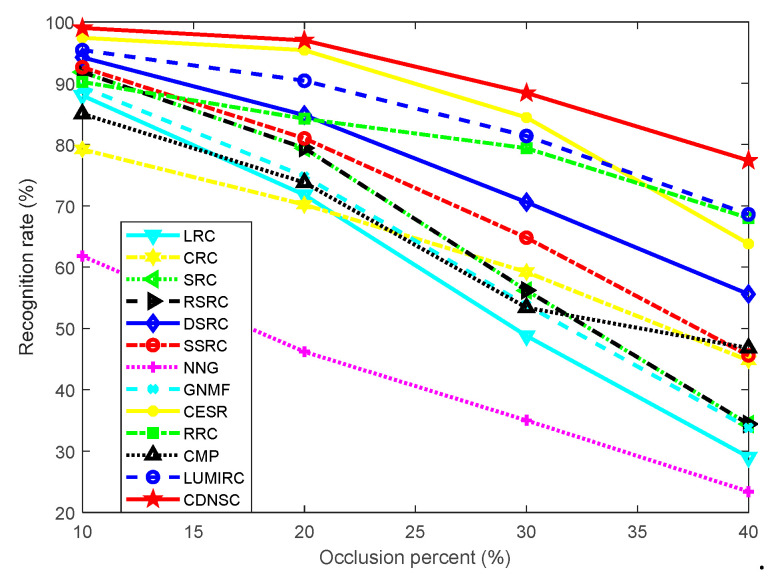
Recognition rate versus the level of camera lens occlusion.

Now, we consider another probable occlusion, continuous camera lens pollution, which often appears in contact-based acquisition. In this experiment, we fix the sample dimension as 40 × 40, and the first three samples of each subject in the dictionary set of the PolyU database are all recruited to compose the dictionary ***A***. The recognition rates of all the methods are displayed in Figure 9.

Obviously, CDNSC outperforms the other compared methods in all occlusion cases. CESR and LUMIRC continue to perform considerably. However, when the occlusion level increases, RRC begins to surpass them. The presence of occlusion misleads CMP from selecting correct dictionary atoms, which leads to its poor performance. CDNSC showed its robustness to scar occlusion on the irregular CASIA database. We believe that it is capable to harness the same case on the more regular PolyU database and will not consider the scar occlusion in this part.

#### 5.3.2. Training Sample Number

**Figure 10:**
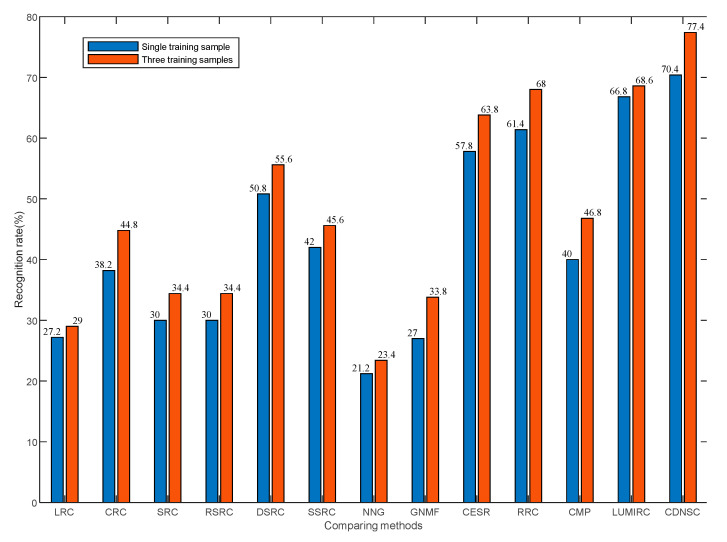
Recognition rate versus the training sample number.

Figure 6a reveals that the variation of sample dimension dose little effect on the palmprint recognition rate. So, we merely pay attention to the impact of the training sample number here. Different from the experiments performed on the CASIA database, we impose 40% camera lens occlusion on the query samples when the training sample number varies. By fixing the sample dimension as 40 × 40, we select the first sample and the first three samples of each subject in the dictionary set to compose dictionary ***A***, respectively. The results are shown in Figure 10.

**Figure 11:**
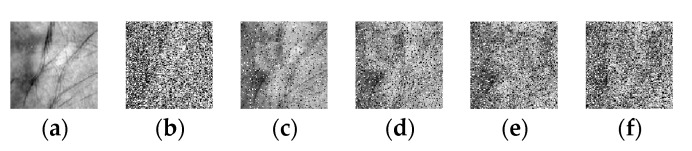
Query samples with dense corruption and the mixed-contaminations in the test set of the PolyU database. (**a**) The original sample, (**b**) Sample with 50% corruption, (**c**–**f**) Samples with 10%–40% mixed-contaminations.

The pixel value of the simulated camera lens pollution is quite close to the palmprint pixel value, which brings extra difficulty to the error detector and corrector in contrast with the scar occlusion (see Figure 8 and Figure 11). As shown in Figure 10, nearly all the methods lost the good performance they ever presented with respect to the scar occlusion, and become sensitive to the camera lens occlusion. To our relief, CDNSC is more robust than the other methods whether with one or three training samples.

#### 5.3.3. Dense Corruption and the Mixed-Contaminations

Finally, we discuss the robustness of CDNSC regarding dense corruption and the mixture of corruption and camera lens occlusion. The sample dimension and assembling processes of the dictionary ***A*** are the same as that in Section 5.3.1. We directly consider the 50% corruption and simulate the mixture case by combining the camera lens occlusion and corruption (level varies from 10% to 40%). The two kinds of contaminations are exhibited in Figure 11. Table 2 displays the recognition rates of all the methods where the best recognition rate of each case is bold.

From Table 2, CESR is considerably robust against dense corruption, while CDNSC and LUMIRC also show promising performance due to their error correctors. However, CDNSC achieves a higher recognition rate of 97.6% than CESR and LUMIRC. Since the mixed-contaminations doesn’t follow the Laplacian or Gaussian distribution, both SRC and RSRC lost their robustness. Although DSRC and SSRC can harness slight mixed-contaminations, they have limited capacity to handle more severe cases. NNG and GNMF are unable to extract robust features, so their performance is rather poor. CESR, RRC, CMP, and LUMIRC show relatively satisfactory results due to their error detectors. However, they are sensitive to the gradually deteriorative mixed-contaminations. Benefitting from the cooperation between CEE and DNSR, CDNSC achieves better results in all contamination levels.

### 5.4. Comparison of Running Times

Apart from recognition rate, computational consumption is another important indicator to evaluate the palmprint recognition methods. This subsection is organized to investigate the efficiency of CDNSC and the other competing methods. For the experiments performed in Section 5.2 and Section 5.3, we specifically consider 40% occlusion and 40% mixed contamination and give the average running time of recognizing a query sample in the two cases. The experimental settings, including sample number, sample dimension, and parameters, follow these given in the previous cases. The comparison among all the competing methods regarding the two cases on the CASIA database and PolyU database is listed in Table 3.

On the whole, the traditional methods, including LRC, CRC, SRC, RSRC, DSRC, SSRC, NNG, and GNMF, take less computation time than the state-of-the-art robust methods, including CESR, RRC, CMP, LUMIRC, and CDNSC. This is because the traditional methods can achieve batch-wise recognition by matrix-based computation. By contrast, the state-of-the-art robust methods have an additional stage to respectively learn a tailored weighted image for each query sample, thus they have to recognize the query samples one by one. Moreover, since the robust methods usually have more than one variable due to their complicated robust models, their optimization processes are consequently more time-consuming, based on the iteratively reweighted optimization strategy. However, the state-of-the-art robust methods present significantly higher accuracy than the traditional methods. From Table 3, we can conclude that CDNSC and CESR achieve a better tradeoff between accuracy and efficiency than the other methods, but CDNSC has a higher accuracy than CESR in all cases (see Figure 7, Figure 9, Table 1 and Table 2).

### 5.5. Multispectral Contactless and Contact-Based Palmprint Recognitions

This subsection is organized to investigate the effectiveness of E-CDNSC. Based on the well-designed objective function (53), CPSO searches such a group of fusion coefficients that manifest the informative spectral features and suppress the less useful spectral features. Figure 3 shows that CDNSC is capable of extracting significant stable features from the seriously contaminated samples. So, it’s reasonable to suppose that the fusion coefficients learned on the original samples are appropriate for fusing the robust features extracted from the multispectral samples.

**Figure 12:**
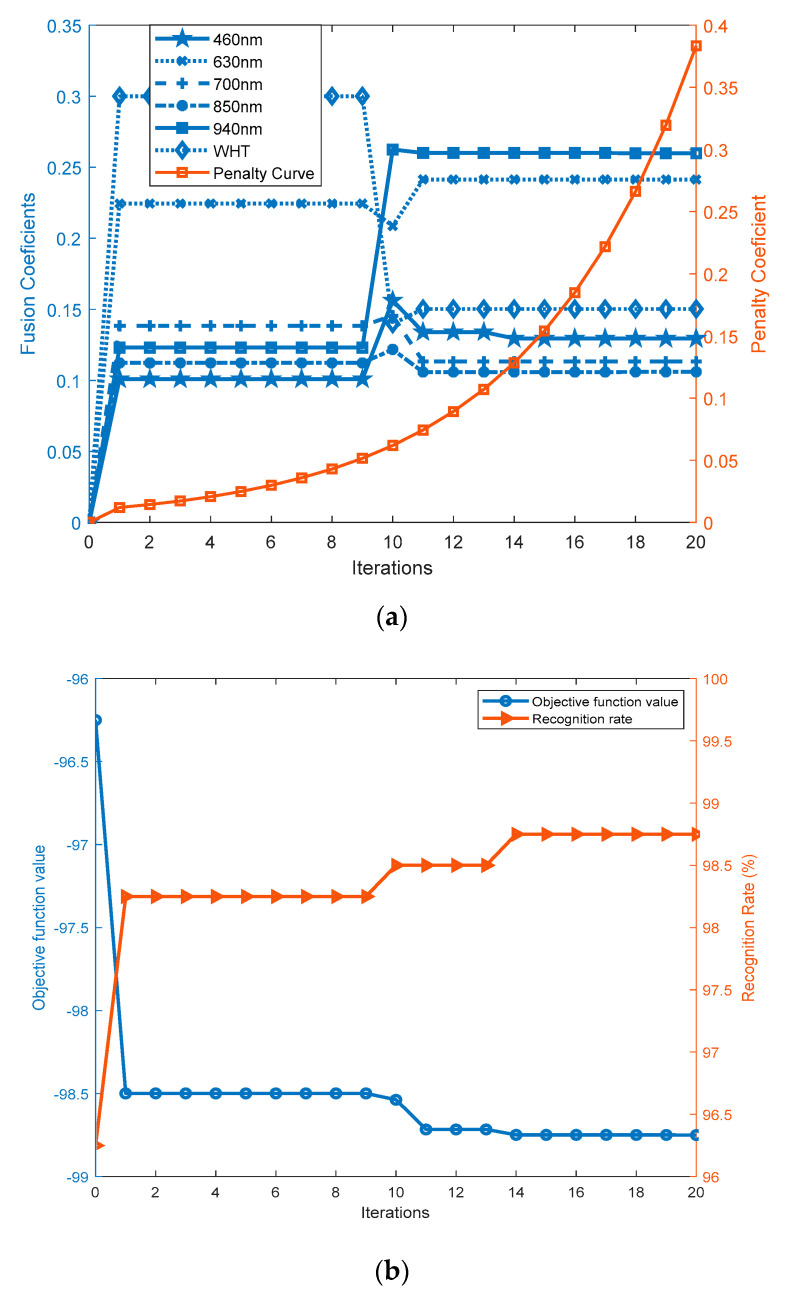
The fusion parameter searching processes. (**a**) Curves of the fusion parameters and penalty parameter, (**b**) Curves of the objective function value and recognition rate.

In the experiments, the sample dimension is fixed as 40 × 40. The first three spectral samples of each subject in the dictionary set of the CASIA database are used to compose the spectrum-dependent dictionary ***A***. The spectral samples in the feature fusion training set are employed to extract spectral features, which are used to train CPSO to obtain the feasible fusion parameters. Given Q=20 (individual number of the particle swarm) and S=6 (particle swarm dimension or spectrum number), the fusion parameter searching processes are shown in Figure 12. 

We set the origin of the coordinate system as the initial positions of the fusion parameters and zero as the initial value of the penalty parameter. Figure 12a indicates that under the punishment of η, CPSO constantly produces new particle swarm $P^{m}\in {\mathbb{R}}_{}^{Q\times S}$ to randomly fly in the defined region until the termination criterion is reached. Obviously, each row of ***P*** is a potential solution to (53). It can be observed that CPSO converges with only 20 iterations. Note that minimizing the constrained objective function (53) is equivalent to maximize the recognition rate function (52). To intuitively present the comparison between multispectral palmprint recognition and single-spectrum palmprint recognition, we define the best single-spectrum recognition rate 96.25% as the initial value of the function (52) and its opposite value −96.25% as the initial value of the function (53). Figure 12b reveals that multispectral palmprint recognition receives more admirable results with a 98.75% recognition rate.

Fixing all the above experimental settings, we now conduct multispectral palmprint recognition based on the learned fusion parameters (see Figure 12a). Four kinds of cases, including illumination and pose variation, 40% scar occlusion, 50% corruption, and the mixed-contaminations with 40% corruption and 40% scar occlusion, are all considered. Similarly, we perform the multispectral palmprint recognition on the PolyU database, where the mixed-contaminations is simulated with 40% corruption plus 40% camera lens occlusion. The results on the two databases are respectively displayed in Table 4 and Table 5, where all the single-spectrum palmprint recognition results are also listed for intuitive comparison.

The experimental results in Table 4 and Table 5 reveal that E-CDNSC can further improve the recognition rate based on the robustness of CDNSC. We also conclude that the fusion parameters learned on the original samples are applicable to fuse the robust features extracted from the contaminated samples. This owes to the flexibility and robustness of CDNSC.

## 6. Conclusions

Considering the robust palmprint recognition, the coding errors caused by contaminations such as gross occlusion, dense corruption, and a mixture of them are insightfully studied in this paper. We combine a correntropy-induced error detector and a sparse error corrector to propose the cooperative error estimator CEE. Moreover, DNSR is designed to encourage the nonnegativity and sparsity of the coefficients. By combining CEE and DNSR, a joint CDNSC is proposed to flexibly handle various contaminations. On the basis, we propose E-CDNSC for multimodal palmprint recognition by introducing a novel CPSO. The correntropy metric function is approximated with a weighted least square formula, while the nonnegative constraint problem is converted into a promising equality constraint. With some skillful techniques, the reformulated problem is effectively optimized via a reweighted ADMM. Extensive experimental results on two public benchmarks reflect the flexibility and robustness of the proposed methods.

Our research reveals the importance of handling the coding errors and the importance of a proper regularizer on precise error estimation. These factors are all vital to protect the flexibility and robustness of recognition methods, when facing various complicated scenarios. This paper only focuses on robust palmprint recognition. However, the active ideas in CDNSC and E-CDNSC can be applied to the other single-mode biometric recognition or multimodal biometric recognition.

## Figures and Tables

**Table 1:** Recognition rates (%) of all the methods with respect to the two kinds of contaminations.

Method	Corruption (50%)	Mixture (10%)	Mixture (20%)	Mixture (30%)	Mixture (40%)
LRC	34.5	79.75	59	27	8.5
CRC	3.5	27	11	4.25	3
SRC	43.75	85.25	67.5	37	12
RSRC	48.25	86	68.75	40.5	19.75
DSRC	43	81.75	65.75	47.25	24
SSRC	53.25	83.25	69.5	52.75	28
NNG	6.25	55.75	22	8.25	3.75
GNMF	18	72.5	43.25	16.75	7.25
CESR	83	92.75	88.75	78.25	55.75
RRC	62.5	74.25	63.25	58	50.75
CMP	38.5	91.75	85.5	76.75	34
LUMIRC	90	92	87.5	80.5	68.5
CDNSC	**94.75**	**94.5**	**90.5**	**85**	**75.25**

**Table 2:** Recognition rates (%) of all the methods with respect to the two kinds of contaminations.

Method	Corruption (50%)	Mixture (10%)	Mixture (20%)	Mixture (30%)	Mixture (40%)
LRC	27	85.2	55.8	19.6	5.8
CRC	3.8	16.8	6.8	2.6	1.4
SRC	51.8	92.2	79.2	43	13.4
RSRC	56.4	92.8	80.4	46.2	20.2
DSRC	46.4	91.8	77.6	47.6	14.4
SSRC	59.6	93	81.6	56.6	23.8
NNG	5.6	50.6	21.6	6.4	2.8
GNMF	11.8	79.8	39.6	13.4	3.6
CESR	90.2	97.8	93.2	80	52.2
RRC	58.6	73.2	55.6	44.6	37
CMP	43	97	92.8	82	38.6
LUMIRC	94.2	94.2	88.6	76.2	63
CDNSC	**97.6**	**97.8**	**95.2**	**86.5**	**75.4**

**Table 3:** Average running time (Seconds) of all the methods regarding the two contaminations.

Method	CASIA Database		PolyU Database	
	Palm Scar Occlusion (40%)	Mixture (40%)	Camera Lens Occlusion (40%)	Mixture (40%)
LRC	0.0002885	0.0002950	0.001936	0.002198
CRC	0.0001210	0.0001185	0.0007160	0.0007155
SRC	0.0498	0.0702	0.1723	0.1932
RSRC	0.1963	0.1997	0.2881	0.2983
DSRC	0.0005825	0.0005855	0.004967	0.005208
SSRC	0.07915	0.0894	0.2135	0.2192
NNG	0.7897	0.9998	12.9199	16.7413
GNMF	0.02232	0.02551	0.1554	0.1582
CESR	0.1939	0.1953	0.5578	0.4518
RRC	0.5808	1.2743	4.1873	8.1859
CMP	0.9250	0.9514	3.1124	3.2647
LUMIRC	0.6391	0.5990	2.6096	2.6455
CDNSC	**0.4452**	**0.4484**	**2.3214**	**2.5753**

**Table 4:** Recognition rates (%) of multispectral and single-spectrum palmprint recognition on the CASIA database.

Spectrum	Pure	Occlusion (40%)	Corruption (50%)	Mixture (40%)
460	95.5	77	94.75	75.25
630	95	73.75	94.5	71.25
700	93.25	68.5	92.25	68.25
850	93.5	71.25	92	70.75
940	96.25	77.25	94.75	74.5
WHT	93.75	75	93.25	72
Multi-spectrum	**98.5**	**89.25**	**97.75**	**85.75**

**Table 5:** Recognition rates (%) of multispectral and single-spectrum palmprint recognition on the PolyU database

Spectrum	Pure	Occlusion (40%)	Corruption (50%)	Mixture (40%)
Blue	99.25	77.4	97.6	75.4
Green	97.8	76	95.8	73.2
Nir	98.8	78.8	96.8	73.8
Red	97.6	75.4	96.2	72.2
Multi-spectrum	**99.8**	**91.2**	**99.2**	**87.8**

## Acronym Definitions

**Acronym**	**Definition**	**Acronym**	**Definition**
ADMM	Alternating direction method of multipliers	KKT	Karush-Kuhn-Tucker
CDNSC	Correntropy-induced Discriminative nonnegative sparse coding	LAR	Least angle regression
CEE	Cooperative error estimator	LRC	Linear regression classifier
CESR	Correntropy-based sparse representation	LUMIRC	Laplacian-uniform mixture driven iterative robust coding
CIM	Correntropy-induced metric	MSE	Mean squared error
CMP	Correntropy matching pursuit	NMF	Nonnegative matrix factorization
CPSO	Constrained PSO	NMR	Nuclear norm-based matrix regression
CRC	Collaborative representation classifier	NNG	Nonnegative garrote
DNSR	Discriminative nonnegative sparse regularizer	PDF	Probability densities functions
DSRC	Discriminative SRC	RRC	Regularized robust coding
E-CDNSC	Extended CDNSC	RSC	Robust sparse coding
ECP	Equality constraint problem	RSRC	robust SRC
GNMF	Graph regularized nonnegative matrix factorization	SRC	Sparse representation classifier
HQ	Half-quadratic	SSRC	Superposed SRC
ICP	Inequality constraint problem	TPNMF	Topology-preserving nonnegative matrix factorization

## Appendix A

### Proof of Proposition 1

**Proof.** It can be easily verified that function $\phi \left(e_{i}\right)=1-I_{\sigma }\left(e_{i}\right)$ satisfies all the conditions of (2.1) in [54]. According to (2.3) in [54], there exists a convex dual function $\psi \left(w_{i}\right)$ such that:

(A1) $$\phi \left(e_{i}\right)=\underset{w_{i}\in \mathbb{R}}{\inf}\left\{\frac{1}{2}w_{i}e_{i}^{2}+\psi \left(w_{i}\right)\right\}$$

when $e_{i}\neq 0$, the infimum of (16) is reached at:

(A2) $$w_{i}=\frac{{\dot{\phi }}_{\sigma }\left(e_{i}\right)}{e_{i}}=\frac{I_{\sigma }\left(e_{i}\right)}{\sigma^{2}}$$

where $I_{\sigma }\left(e_{i}\right)=\exp\left(-e_{i}^{2}/2\sigma^{2}\right)$. □

## Appendix B

### Proof of Lemma 1

**Proof.** As the value $t^{*}$ is a local minimum of (22), then $\left(t^{*},z^{*}\right)$ is a local minimum of (23). Apparently, the functions f(t) and h(t,z) in (23) are also continuously differentiable. Once ∇g(t) is linearly independent, ∇h(t) is linearly independent, too. For (23), according to the first-order Lagrange necessary condition of the ECP(proposition 3.1.1 in [55]), there exists a unique value $\varphi^{*}$ such that:

(A3) $$\{\begin{array}{c} \nabla_{t}L\left(t^{*},z^{*},\varphi^{*}\right)=\nabla_{t}f\left(t^{*}\right)+\varphi^{*}\nabla_{t}h\left(t^{*},z^{*}\right)=0\\ \nabla_{z}L\left(t^{*},z^{*},\varphi^{*}\right)=-2\varphi^{*}z^{*}=0. \end{array}$$

If $g\left(t^{*}\right)\neq 0$, we can obtain $z^{*}=\sqrt{g\left(t^{*}\right)}\neq 0$. From (A3), we get:

(A4) $$\varphi^{*}=0\left(g\left(t^{*}\right)\neq 0\right)$$

For (23), according to the second-order Lagrange necessary conditions of the ECP (3.1.1 in [55]), we have:

(A5) $$\left[\begin{array}{cc} \omega & \varpi \end{array}\right]\left[\begin{array}{cc} \nabla_{tt}^{2}L\left(t^{*},z^{*},\varphi^{*}\right) & 0\\ 0 & -2\varphi^{*} \end{array}\right]\left[\begin{array}{c} \omega \\ \varpi \end{array}\right]\geq 0$$

For arbitrary ω and $\varpi \in {\mathbb{R}}^{1}$, they always satisfy:

(A6) $$\left[\begin{array}{cc} \nabla_{t}L\left(t^{*},z^{*},\varphi^{*}\right) & \nabla_{z}L\left(t^{*},z^{*},\varphi^{*}\right) \end{array}\right]\left[\begin{array}{c} \omega \\ \varpi \end{array}\right]=\nabla_{t}g\left(t^{*}\right)\omega -2z^{*}\varpi =0$$

The Lagrange multiplier theorem of the ECP reveals that $\varphi^{*}$ exists uniquely, for which the conclusions drawn under specific situations reflect the truth. We can obtain desired results by substituting differently defined ϖ into (A6) as long as it satisfies (A7). We first define:

(A7) $$\varpi =\{\begin{array}{cc} 0 & g\left(t^{*}\right)=0\\ \frac{\nabla_{t}g\left(t^{*}\right)\omega }{2z^{*}} & g\left(t^{*}\right)\neq 0 \end{array}$$

With (A4) and (A7), we say that whether $g\left(t^{*}\right)=0$ or not, $\varpi \varphi^{*}\varpi =0$ always holds. For ω satisfying $\nabla_{t}g\left(t^{*}\right)\omega =0$ $\left(g\left(t^{*}\right)=0\right)$, we obtain the second-order necessary condition of (23):

(A8) $$\omega \nabla_{tt}^{2}L\left(t^{*},z^{*},\varphi^{*}\right)\omega \geq 0$$

By redefining $\omega =0,\varpi \neq 0\left(g\left(t^{*}\right)=0\right)$, we obtain $-2\varphi^{*}\varpi^{2}\geq 0$ according to (A6). That is to say

$$\varphi^{*}\leq 0\left(g\left(x^{*}\right)=0\right)$$

Finally, we obtain (24) by combining (A3), (A4), and (A9). □

## Appendix C

### Proof of Proposition 2

**Proof.** Note $\bar{ℒ}\left(\tilde{e},B,u,x,\phi_{1},\phi_{2},\phi_{3},\rho \right)$ in (33) can be rewritten as $\bar{ℒ}\left(W,e,B,u,x,\phi_{1},\phi_{2},\phi_{3},\rho \right)$. Since the convergence properties of ADMM have got in-depth research in [56], when $W^{k}$ is fixed the updated sequence in Algorithm 1 always satisfies $\bar{ℒ}\left(W^{k},x^{k-1},e^{l},B^{l},u^{l},\phi_{1}{}^{l},\phi_{2}{}^{l},\phi_{3}{}^{l},\rho^{l}\right)\geq \bar{ℒ}\left(W^{k},x^{k},e^{l},B^{l},u^{l},\phi_{1}{}^{l},\phi_{2}{}^{l},\phi_{3}{}^{l},\rho^{l}\right)$.

When $x^{k}$ is fixed, we have..according to Proposition 1. Hence, the sequence $\bar{ℒ}\left(W^{k},x^{k},e^{l},B^{l},u^{l},\phi_{1}{}^{l},\phi_{2}{}^{l},\phi_{3}{}^{l},\rho^{l}\right)$ generated by CDNSC is non-increasing. By studying the properties of the correntropy [40] and formula (27), it is clear that J(W,e,x,z) has a lower bound. In conclusion, CDNSC converges. □
